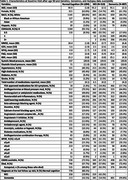# Leveraging the Alzheimer’s Disease Research Center Data to Close the Knowledge Gap about Cognitive Resilience and Frailty in Individuals Age 90 or Older

**DOI:** 10.1002/alz70861_108890

**Published:** 2025-12-23

**Authors:** Mary Sano, Carolyn W. Zhu, John F. Crary, Douglas R. Galasko, Alison M. Goate, Claudia H. Kawas, Paul O'Reilly

**Affiliations:** ^1^ Icahn School of Medicine at Mount Sinai, New York, NY USA; ^2^ James J. Peters VA Medical Center, Bronx, NY USA; ^3^ UCSD, San Diego, CA USA; ^4^ Institute for Memory Impairments and Neurological Disorders (UCI‐MIND), University of California, Irvine, Irvine, CA USA

## Abstract

**Background:**

The US population aged 90 or older is growing faster than other groups. Yet they are often excluded from research, including clinical trials. It is imperative that we understand cognitive resilience and frailty in this group. Since 2005, the National Institutes of Health (NIH)/National Institute on Aging (NIA) funded Alzheimer’s Disease Research Center (ADRC) program has collected standardized longitudinal clinical data using the Uniform Dataset (UDS) and genetic and biomarker data on participants with normal cognition, Mild Cognitive Impairment (MCI) or dementia. Here we describe data and sample availability in this long‐standing project to assess potential to examine cognitive health in those over 90.

**Method:**

Data were drawn from the UDS (up to March 2024 data freeze, N=50,259). Individuals who had at least 2 visits after age 90y were included. The first such visit was defined as baseline. Using consensus diagnosis determined from clinician judgment, neuropsychological testing, and standardized scales such as the CDR at baseline, participants’ cognitive status were grouped into cognitively normal (CN, N=1294), MCI (*N* =519) and dementia (*N* =807).

**Result:**

Characteristics of the sample at baseline visit after age 90y, APOE genotype, availability of genomics and autopsy data by cognitive status are presented in Table 1. Mean age was 91y, 60.8% were female. Mean±SD MMSE was 28.4±1.8, 26.3±2.7, and 19.3±6.4 for CN, MCI and dementia, respectively. Participants were followed for an average of 3.5±2.5, 2.6±1.7, and 2.3±1.5 years after baseline. At their last follow‐up visit, among those who were baseline CN, 60.1% remained CN, 19.9% progressed to MCI, and 16.4% to dementia. Among baseline MCI, 54.9% progressed to dementia. Dementia diagnosis remained for 99.8% of baseline dementia cases. 746 CN (57.7%), 300 MCI (57.8%), and 584 dementia (72.4%) cases had died. Among those who died, 515 (69.0%), 187 (62.3%), and 376 (64.4%) were autopsied. APOE genotypes will be used to examine the relationship between APOE e4 and cognitive trajectories over time. GWAS data for 938 (72.5%), 349 (67.2%), and 498 (61.7%) cases are available for more in‐depth analyses.

**Conclusion:**

The availability of genetics and autopsy data enables understanding cognitive resilience and frailty in individuals age 90y or older.